# COVID-19 and Its Effects on the Hepatobiliary System: A Literature Review

**DOI:** 10.7759/cureus.80231

**Published:** 2025-03-07

**Authors:** Sariya Khan, Jumana Hussain Timraz, Nourah A Al Ghamdi, Nada Y Metwali, Faten A Yaseen, Albatool M Alshaqha, Sarah H Alamri, Heba Turkistani, Anas Dwaima, Ibraheem Ali Algarni

**Affiliations:** 1 General Medicine and Surgery, Batterjee Medical College, Jeddah, SAU; 2 Medicine and Surgery, Batterjee Medical College, Jeddah, SAU; 3 Medicine and Surgery, Fakeeh College of Medical Sciences, Jeddah, SAU; 4 Obstetrics and Gynecology, Batterjee Medical College, Jeddah, SAU; 5 Internal Medicine, Batterjee Medical College, Jeddah, SAU; 6 Internal Medicine, International Medical Center Hospital, Jeddah, SAU; 7 Family Medicine, Faculty of Medicine in Rabigh, King Abdulaziz University, Jeddah, SAU

**Keywords:** alanine aminotransferase (alt), alkaline phosphatase (alp), angiotensin-converting enzyme 2 (ace2), aspartate aminotransferase (ast), chronic liver disease, covid-19, covid vaccine, liver, sars-cov-2

## Abstract

COVID-19 encompasses a wide clinical spectrum, from mild influenza-like illness to severe pneumonia and systemic complications. There is emerging literature on hepatobiliary involvement in COVID-19, especially elevation in liver enzymes as surrogate markers of liver injury. Angiotensin-converting enzyme 2 receptors within the hepatobiliary system are a portal of entry for SARS-CoV-2, after which injury may be perpetuated through hypoxia and cytokine storms. This literature review covers studies published before 2024 from databases such as PubMed, Google Scholar, Springer, and BMC Library. The keywords used were "COVID-19", "liver", "SARS-CoV-2", "chronic liver disease", and other relevant terms to ensure a wide scope of investigation. The most common liver enzymes elevated among COVID-19 patients include aspartate transaminase, alanine transaminase, and alkaline phosphatase, all of which are associated with the severity of the disease. Chronic liver disease (CLD) and hepatocellular carcinoma (HCC) patients have worse outcomes with increased ICU admission rates and increased mortality. COVID-19 vaccination in CLD and liver transplant recipients is very often associated with suboptimal antibody responses, adding to the risks. SARS-CoV-2 causes liver involvement through direct viral cytopathic effects, immune-mediated injury, and systemic hypoxia. Individuals with CLD are particularly vulnerable to severe illness.

## Introduction and background

The COVID-19 pandemic, caused by the novel SARS-CoV-2, has become one of the most important global health crises in recent times. While initially recognized based on respiratory manifestations, COVID-19 is increasingly being appreciated as a systemic illness with widespread effects on multiple organ systems, including but not limited to the cardiovascular, renal, gastrointestinal, and hepatobiliary systems [1]. The liver and biliary tract are increasingly being recognized to be susceptible to both direct and indirect effects of SARS-CoV-2. Abnormalities in liver enzymes have been observed in 14% to 53% of hospitalized patients with COVID-19, hence the involvement of the hepatobiliary system. Furthermore, it appears that patients with pre-existing liver disease or metabolic disorders at the time of infection are more susceptible to adverse outcomes [2]. Despite these observations, the exact mechanisms for hepatobiliary dysfunction in COVID-19 are incompletely understood and probably multifactorial, including direct viral cytotoxicity, immune-mediated injury, drug-induced liver injury, and ischemia-related damage [2,3]. Due to the nature of the virus, it causes human-to-human transmission via respiratory droplets, close contact, or by touching contaminated surfaces, thus causing rapid spread of the virus and increasing the number of cases [4]. SARS-CoV-2 is a β-coronavirus that is an enveloped, non-segmented, positive-sense RNA virus. The virus is now known to use angiotensin-converting enzyme 2 (ACE2), found in the lower respiratory tract, to infect humans [5]. Despite COVID-19 being a respiratory disease, some studies have shown its effects on other organs, including the heart, gastrointestinal tract, and liver [6].

This review aims to explore the complex relationship between COVID-19 and the hepatobiliary system. We will further discuss pathophysiology, clinical manifestations, and diagnostic findings related to liver and biliary tract involvement. Further, the paper discusses the COVID-19 experience in patients with pre-existing liver disease and the hepatic consequences of the therapeutic interventions practiced. This review synthesizes current information to advance knowledge regarding the hepatobiliary complications of COVID-19 and creates a foundation by which patient care can be optimized and further research can continue.

## Review

Materials and methods

This literature review included many published studies up to 2024. The databases used include Google Scholar, PubMed, Springer, and BMC Library using the following keywords: “liver", "COVID-19", COVID vaccine", "SARS-CoV-2", chronic liver disease", angiotensin-converting enzyme 2 (ACE2)", "aspartate aminotransferase (AST)", "alanine aminotransferase (ALT)", and "alkaline phosphatase (ALP)”. This review included studies in English that were central to the theme of this study. Articles in languages other than English were excluded. Data extraction was applied to discern patterns, trends, and key findings, contributing to a comprehensive understanding of the subject.

Pathophysiology of SARS-CoV-2 and hepatobiliary involvement

The involvement of the hepatobiliary system in COVID-19 is underpinned by complex, multifactorial mechanisms that include direct viral effects, immune dysregulation, and systemic consequences of critical illness. Understanding these mechanisms provides critical insights into the disease's impact on the liver and biliary system. ACE2 is highly expressed in the liver, cholangiocytes, and other tissues and is considered the primary entry point of SARS-CoV-2 into human cells [7,8]. Cholangiocytes are indispensable in bile secretion and liver homeostasis and have been identified as one of the most vulnerable targets for SARS-CoV-2 infection. Thus, the direct viral invasion of such cells may lead to hepatobiliary dysfunction. Indeed, most patients with severe COVID-19 have exhibited an exaggerated immune response as defined by the high level of pro-inflammatory cytokines such as IL-6, TNF-α, and IFN-γ in what is sometimes called the cytokine storm [9]. This hyperinflammatory state may also be responsible for diffuse tissue injury, including immune-mediated hepatocellular injury. The mechanisms of COVID-19-associated hypoxemia and systemic inflammation may cause ischemic hepatitis, or so-called "shock liver," as hepatic blood flow has been reduced because of hemodynamic instability in most patients in the severe stage of this illness [10]. COVID-19 pertains to the hypercoagulable state that increases microvascular thrombosis in the liver. These events may promote further sinusoidal congestion and hepatocellular injury. Evidence has shown a correlation between coagulation and liver damage in COVID-19, highlighted by the correlation of transaminase with coagulopathy, as evidenced by laboratory markers like prothrombin time, international normalized ratio, fibrinogen, D-dimer, fibrin/fibrinogen degradation products, and platelet count. Low platelet count, slightly raised prothrombin time, and raised D-dimer are typical laboratory features of critically ill SARS-CoV-2 patients, termed "COVID-19-associated coagulopathy." These abnormalities are linked with poor outcomes. Medications commonly utilized to manage COVID-19 include antiviral agents, such as remdesivir, and immunomodulators, such as tocilizumab and corticosteroids, all associated with liver injury [11]. Though drug-induced liver injury is not related to a pathophysiological mechanism by the virus, it accounts for a significant percentage of the hepatobiliary complications related to COVID-19. SARS-CoV-2 also affects the gastrointestinal tract, which interferes with the gut-liver axis: dysbiosis and an increase in intestinal permeability may give rise to a translocation of microbial products, including lipopolysaccharides, capable of further enhancing liver inflammation and injury [12,13].

Clinical characteristics of COVID-19 and its impact on individuals with healthy livers

COVID-19 is typically characterized by the symptoms of viral pneumonia, such as fatigue, fever, dry cough, headache, and anosmia, which may evolve into respiratory failure [14,15]. The pathogen displays a wide range of severity, causing difficulty in determining infection effects [16,17]. Patients with COVID-19 commonly exhibit elevated markers associated with liver injury, such as ALT, AST, gamma-glutamyltransferase (GGT), and ALP [18-20]. Sequencing studies have revealed ACE2 microRNA expression in a subpopulation of cholangiocytes but no or minimal expression in hepatocytes or other liver cell types [21,22]. These findings strongly suggest that SARS-CoV-2 doesn’t directly cause cytopathic liver damage. The cytokine and hypoxia storm of SARS-CoV-2 could be the causative element in liver injury, which is associated with multi-organ failure in some patients [23,24]. The cytokine storm may also be implicated in the disseminated intravascular coagulation (DIC) and thrombocytopenia, which can worsen the DIC complications, as observed in COVID-19 [25]. One report demonstrated that, in contrast to patients with mild COVID-19, patients with non-COVID-19 pneumonia didn’t exhibit elevated levels of AST, ALT, or GGT [26], suggesting that patients with mild COVID-19 may have elevated liver damage markers independently of the presence of inflammation. However, COVID-19 doesn’t affect healthy livers as much as it affects patients with multiorgan failure and severe infections [27,28].

Impact of COVID-19 on chronic liver diseases and hepatocarcinoma

Non-alcoholic Fatty Liver Disease and Hepatic Cirrhosis

Patients with either non-alcoholic fatty liver disease (NAFLD), especially those who suffer from diabetes mellitus and obesity [29], or hepatic cirrhosis are at risk of moderate or severe COVID-19 infection, yet COVID-19 causes a progression of liver injury by direct and indirect mechanisms. A direct mechanism occurs when SARS-CoV-2 binds to hepatic cells through the interaction between spike glycoprotein S and ACE2 receptors, followed by viral replication. However, this direct mechanism does not lead to liver injury progression in NAFLD since there is no increased uptake of the COVID-19 virus by the liver due to the absence of alterations in the expression of hepatic genes in SARS-CoV-2 [30]. While in hepatic cirrhosis, most of the hepatocytes within cirrhotic nodules express ACE2 and increased circulating ACE2 and angiotensin II levels, which aid SARS-CoV-2 in entering the target cells [31]. The cytokine storm mechanism is induced by releasing multiple proinflammatory cytokines and high serum levels of inflammatory markers, including IL-6, IL-8, IL-17, IL-1B, and IFN-γ [32]. Nonetheless, in cirrhosis, pancytopenia occurs as the result of disturbance in the reticuloendothelial system, leading to impairment of immune protein synthesis, loss of immune surveillance activities, immune cell dysfunction, and circulating immune cell damage; that is how liver cirrhosis is connected to COVID-19 [29,33]. Drug-induced liver injury is one more mechanism in progressing NAFLD and hepatic cirrhosis; applying antiviral medications (e.g., remdesivir, lopinavir/ritonavir, acetaminophen, and hydroxychloroquine), antibiotics, and corticosteroids for treating COVID-19 causes a concern about inducing hepatotoxicity as they are associated with an elevation of ALT and AST [29].

Viral Hepatitis

Chronic liver diseases (CLD), including cirrhosis and hepatocellular carcinoma (HCC), are largely caused by the hepatitis B virus (HBV), followed by the hepatitis C virus (HCV). The present global prevalence of CLD accounts for about 1.5 billion cases, 28% of which are due to HBV and 9% to HCV [34]. Since COVID-19 has shown evidence of disturbing the levels of liver enzymes and might trigger some degree of liver injury, it is important to know the extent of the disease’s severity and risks in HBV/HCV patients superimposed with SARS-CoV-2 infection. However, drawing a consistent and reliable theory is challenging as there hasn’t been enough research and data, possibly due to the low numbers presenting with such coinfection. For instance, in one of the cohort studies with 5700 COVID-19 patients, only eight of them had underlying HBV and three with HCV [35]. Furthermore, it is assumed that the level of liver injury during a SARS-CoV-2 infection is almost equal to the one with an underlying HBV-CLD. In a 326-case study based in the COVID-19-designated hospital in China (20 of 326 had had HBV), it was reported that the degree of liver injury does not worsen by the presence of HBV-SARS-CoV-2 coinfection. Plus, there was no major difference in the discharge rate from hospitalization [21]. Additionally, another study that enrolled 105 HBV-CLD patients with COVID-19 had collected their liver function tests, in which 20.9%, 27.6%, and 6.6% had elevated ALT, AST, and bilirubin, respectively [36]. This analysis concludes that HBV-CLD is associated with poor prognosis of COVID-19. Even though there’s some degree of liver injury, it still lies within the same range of hepatic dysfunction caused by SARS-CoV-2 individually. This is proven by a retrospective study based in China in a non-ICU ward: in 79 COVID-19 cases, 31.6%, 35.4%, and 5.1% had elevated ALT, AST, and bilirubin, respectively [37]. Besides, the increased link for worse prognosis in HBV/HCV patients might be due to the usual presence of other comorbidities such as diabetes or cardiovascular diseases [38], which all are common risk factors of COVID-19 [35]. Thus, viral hepatitis can not individually act as a risk factor resulting in poor prognosis with SARS-CoV-2 or an increase in discharge rate after hospitalization.

Hepatocellular Carcinoma

Generally, cancer patients are considered to be at more risk for a severe COVID-19 with poor prognosis and complications. In a nationwide study based in China, it was recorded that cancer patients were most likely to need invasive ventilation and ICU admission and have a higher rate of mortality [39]. Moreover, since HCC is considered the 6th most common cancer with a high mortality rate [40], it is expected that HCC patients are at an increased risk of critical COVID-19 disease. As mentioned earlier, CLD has shown adverse effects when complicated with SARS-CoV-2, let alone the liver injury caused by COVID-19 in isolation. Since most HCCs are caused by cirrhosis and other CLDs that result in an increased risk of COVID-19, likewise, findings show that cancer patients will have deteriorating outcomes [40]. Therefore, it is safe to say that HCC patients are at increased risk and are expected to have worse consequences than non-HCC/SARS-CoV-2 patients.

Abnormal liver test results associated with COVID-19

Several studies have reported liver function test impairment in association with Corona family infections. Recent studies of 11,245 COVID-19 patients suggest abnormal liver enzyme levels. Reported data included AST and ALT in the early stages of COVID-19 patients. Although ALT is a specific liver damage marker, AST changes are nonspecific because of its widespread expression [41].

Another study on 218 patients found that the serum albumin level decreased in 108 (50%), suggesting inflammation and/or liver disease. The incidence of decreased serum albumin level was significantly higher in severe COVID-19 patients than in non-severe patients. For hematology and coagulation: Leukocytes were normal in most patients (74.8%), with 19.2% having a decrease. Lymphopenia was found in 62.0% of patients with liver injury. Other laboratory findings in the liver injury group included high C-reactive protein, a marker of inflammation; high fibrinogen percentage; white blood cell count; and neutrophil, which may be important predictors of liver injury in patients with COVID-19 [42].

In an autopsy investigation, the virus was detected not only in the respiratory system but also in the digestive system. As the viral DNA is detectable in stool samples, the fecal-oral transmission route has been suggested, and it may be responsible for liver involvement. Liver damage can also occur because of systemic respiratory infections because of immune responses [43].

Prognosis and COVID-19 disease severity

A study found that the alveolar arterial blood oxygen tension difference in patients with abnormal liver enzymes was dramatically higher during arterial blood gas tests. However, patients with severe COVID-19 may have an increased risk of developing hypoxic-ischemic liver disease [44]. Males and people with higher levels of leukocytes, neutrophils, C-reactive protein, and CT scan (X-ray CT) lesions are more likely to have hepatic injury. Another study found that COVID-19 with liver injury had higher CT scores and that CT scores were the predictive factor of liver injury in COVID-19. Although it is not known whether changes in lung CT are related to liver injury, patients with severe imaging lesions should be closely monitored for liver damage to initiate early intervention. Additionally, there was an association between liver damage and prolonged hospital stays, so liver damage may impact the prognosis of COVID-19 patients [45]. An elevated AST, ALT, or ALP above the upper limits of normal significantly increased the odds of ICU admission. There was no correlation between high total bilirubin levels, high international normalized ratio levels, or low albumin levels and ICU admission. Yet, patients with hypoalbuminemia at the time of admission were more likely to die, develop at least one episode of hypotension, require intubation, or require vasopressors. However, they also found that there may be certain effects on liver function in COVID-19 patients caused by levels of inflammation within the body [46].

Patients may be predisposed to develop more severe disease and poor prognosis if they suffer from comorbid conditions such as non-alcoholic steatohepatitis (NASH), alcoholic liver disease, and viral hepatitis to some extent when accompanied with diabetes, hypertension, or hepatic cancer. In conclusion, elevated transaminases, hypoalbuminemia, and other hepatobiliary laboratory abnormalities could provide predictors of a more severe disease course, including requiring hospital admission and mortality in COVID-19 patients [3,39]. A better understanding of liver dysfunction may aid the preparation of treatment for patients, as severe COVID-19 infection frequently results in severe liver dysfunction [47].

Analysis of antibody responses after COVID-19 vaccination in liver transplant recipients and those with chronic liver diseases

Patients who are suffering from CLD, such as candidates for liver transplantation (LT), cirrhosis, and immunosuppressed individuals after hepatic transplantation appear to be at increased risk of infections including COVID-19 disease which tend to increase mortality, especially for patient with cirrhosis; therefore, vaccination against SARS-CoV-2 is needed as soon as possible [48]. Immunocompromised patients or other liver transplant recipients weren't included in the registration trials of mRNA vaccine studies for SARS-CoV-2. The data on the outcome of liver transplant recipients with COVID-19 are inconsistent. There's a particular trend toward higher mortality risks in transplant recipients. Although the clinical efficacy of the COVID-19 vaccine in immunocompromised patients was unknown, many societies had recommended the vaccination of this highly vulnerable patient population. It has been suggested, based on circumstantial evidence, that it is prudent to vaccinate immunocompromised subjects since the advantages outweigh the risks. Additionally, a new study reported that only 17% of organ transplant recipients developed a detectable antibody to SARS-CoV-2 spike protein after the 1st dose of mRNA vaccines. A lower immune reaction was anticipated since humoral immunity is critical for antibody response after the vaccination; however, the response seen after the first dose was disappointingly very low. In this prospective study, adult patients with CLD and LT recipients were included, whereas patients with human immune deficiency virus and those with previous exposure to COVID-19 were eliminated. Clinical characteristics were collected, such as age, sex, comorbidities (hypertension, heart failure, chronic kidney diseases), as well as causes of liver disease. Detecting cirrhosis in these cases was done using biochemical or clinical evidence. The types of vaccination and the dates were confirmed in all patients, and all side effects after the first and second doses were collected. They planned to continue following the patient for one year to understand the differential attrition of antibody levels with time. The result was that LT recipients, use of two or more immunosuppression medications and vaccination with a single dose of the Johnson & Johnson vaccine were associated with poor immune response on multivariable analysis. None of the patients suffered serious adverse effects; however, vaccinations caused poor antibody response in 24% of those with CLD and 61% of LT recipients [49]. Table 1 summarizes key findings from various studies.

**Table 1:** Summary of key findings from literature review RCT: randomized controlled trial, COVID-19: coronavirus disease 2019, UK: United Kingdom, GGT: gamma-glutamyl transpeptidase, SARS-CoV-2: severe acute respiratory syndrome coronavirus 2, ADRs: adverse drug reactions, AAC: acute acalculous cholecystitis

Title	Author	Year	Study design	Sample size/population	Key finding
Long-term gastrointestinal and hepatobiliary outcomes of COVID-19: A multinational population-based cohort study from South Korea, Japan, and the UK [50]	Lee et al.	2024	Non-RCT observational study	Total of 10,027,506 Korean, 12,218,680 Japanese, and 468,617 UK patients	COVID-19 patients have a higher risk of gastrointestinal diseases, hepatobiliary diseases, and other digestive abnormalities, with the risk increasing as the severity of the infection increases.
The clinical implication of gamma-glutamyl transpeptidase in COVID-19 [51]	Liu et al.	2021	Non-RCT observational study	66 laboratory-confirmed patients with COVID-19	Elevated GGT levels in COVID-19 patients are common in severe cases and positively associated with prolonged hospital stay and disease severity, making it a potential biomarker for SARS-CoV-2 Infection
Global burden of vaccine-associated hepatobiliary and gastrointestinal adverse drug reactions, 1967-2023: A comprehensive analysis of the international pharmacovigilance database [52]	Lee et al.	2024	Non-RCT observational study	6,842,303 reports of vaccine-associated ADRs	Most vaccine-associated hepatobiliary and gastrointestinal adverse drug reactions occurred within 1 day, emphasizing the need for vigilant monitoring and timely management
Hepatobiliary system and intestinal injury in new coronavirus infection (COVID-19): a retrospective study [53]	Kozlov et al.	2023	Non-RCT observational study	80 patients with COVID-19	Intestinal permeability and microbiota changes are key drivers of gastroenterological manifestations and increased COVID-19 Severity in patients
Acute acalculous cholecystitis as an early manifestation of COVID-19: case report and literature review [54]	D’Introno et al.	2022	Case report	1	AAC is a potential complication of COVID-19, with gastrointestinal symptoms being a potential target of SARS-CoV-2

## Conclusions

COVID-19 infections are not only associated with the typical symptoms of viral pneumonia that evolves to respiratory failure in severe cases, but they can also be associated with hepatobiliary abnormalities due to the presence of ACE2 receptors on cholangiocytes, plus the cytokine and hypoxia storm within the hepatocytes. This makes them the causative element in the liver injury presented in COVID-19 patients, as elevated liver enzymes are ALT, AST, GGT, and ALP. After all, these markers can be used to predict poor prognosis and indicate early intervention since these enzymes have been established as a risk factor for severe COVID-19 in multiple studies. However, COVID-19 doesn’t affect healthy livers as much as it affects patients with CLD and HCC. Finally, those CLD patients superimposed with COVID-19 were found to have low antibody reactions upon receiving the vaccinations, especially the liver transplant recipients, which is less than what is needed to reduce the risks of severe COVID-19 infection.
